# Troponin dependent 30-day mortality in patients with acute pulmonary embolism

**DOI:** 10.1007/s11239-023-02864-0

**Published:** 2023-07-24

**Authors:** Emilie Sonne-Holm, Matilde Winther-Jensen, Lia E. Bang, Lars Køber, Emil Fosbøl, Jørn Carlsen, Jesper Kjaergaard

**Affiliations:** 1 Department of Cardiology, The Heart Centre, Copenhagen University Hospital Rigshospitalet, Blegdamsvej 9, Copenhagen, 2100 Denmark; 2 Department of Clinical Medicine, Faculty of Health and Medical Sciences, University of Copenhagen, Copenhagen, Denmark; 3 Department of Data, Biostatistics and Pharmacoepidemiology, Centre for Clinical Research and Prevention, Bispebjerg and Frederiksberg Hospital, Bispebjerg, Denmark

**Keywords:** Epidemiology, Mortality, Risk assessment, Pulmonary embolism, Troponin

## Abstract

Background: Troponin concentrations above upper reference are associated with increased mortality in patients with pulmonary embolism (PE). We aimed to assess whether risk of 30-day mortality increases in a dose-response relationship with concentration of troponin. Methods: Using Danish national registries, we identified patients ≥ 18 years of age hospitalized with first-time PE between 2013 and 2018 and available troponin measurements − 1/+1 day from admission. Patients were stratified into quintiles by increasing troponin concentration. Risk of 30-day mortality was assessed performing cumulative mortality curves and Cox regression model comparing the troponin quintiles. Results: We identified 5,639 PE patients of which 3,278 (58%) had a troponin concentration above upper reference. These patients were older (74 years), 50% male and with heavier comorbidity compared to patients with non-elevated troponin. We found increasing 30-day mortality with increasing troponin concentration (1% in 1st quintile (95% CI 0.5–1.5%), 2% in 2nd quintile (95% CI 1-2.5%), 8% in 3rd quintile (95% CI 5–9%), 11% in 4th quintile (95% CI 9–13%) and 15% in 5th quintile (95% CI 13–16%), confirmed in a Cox model comparing 1st quintile with 2nd quintile (HR 1.09; 95% CI 0.58–2.02), 3rd quintile (HR 3.68; 95% CI 2.20–6.15), 4th quintile (HR 5.51; 95% CI 3.34–9.10) and 5th quintile (HR 8.09; 95% CI 4.95–13.23). Conclusion: 30-day mortality was strongly associated with troponin concentration useful for improving risk stratification, treatment strategies and outcomes in PE patients.

**Supplementary Information:**

The online version contains supplementary material available at 10.1007/s11239-023-02864-0.

## Introduction

Pulmonary embolism (PE) is the third most common acute cardiovascular condition worldwide [1] with increasing incidence rates [2] ranging from 39 to 115 per 100,000 person years [3]. The condition can be fatal with 30-day mortality reaching as high as 15% [1]. A critical determinant of outcome in acute PE is right ventricular (RV) failure, defined as a rapidly progressive syndrome resulting from impaired RV filling and/or reduced RV flow output due to sudden pulmonary obstruction [4]. Dilatation of the RV eventually impacts the filling of the left ventricle (LV) and coronary perfusion and thus reduces cardiac output leading to systemic hypotension, hemodynamic instability and ultimately death [5]. However, absence of hemodynamic instability does not exclude beginning and/or progressing RV dysfunction, and early risk assessment of PE patients is essential [6].

Troponin I and T (TnI and TnT) serves as markers of myocardial injury caused by ischemic damage, toxic effects, or hemodynamic stress. In patients with PE, 30–60% have elevated levels of troponin TnI and/or TnT [7–10] as a consequence of RV dysfunction. Troponin concentrations above threshold are associated with increased risk of mortality in both unselected PE patients and in those, who are hemodynamically stable at presentation [8]. Troponin positivity in combination with echocardiographic and clinical findings, thus serves as an important tool for early risk assessment and choice of treatment strategy in patients with acute PE [1].

However, with new and promising treatment methods for especially hemodynamic stable PE patients (e.g., low-dose thrombolysis, ultrasound catheter-based thrombolysis) [11–16], optimization of risk stratification is important for early identification of patients at increased risk of hemodynamic deterioration and death.

We therefore aimed to assess (1) the association between concentrations of TnI/TnT above threshold and 30-day all-cause mortality among patients with first-time PE and (2) the proportionality between 30-day all-cause mortality and concentration of TnI/TnT using quintiles.

## Methods

Using the Danish National Patient Register (DNPR), we identified patients ≥ 18 years of age admitted to the hospital with a first-time diagnosis of PE between 1st of January 2013 and 31st of December 2018. In Denmark all citizens have a unique civil registration number, used by all authorities as a number for identification. All hospital admissions, emergency department contacts and outpatient clinic contacts in Denmark since 1977 are covered in the DNPR. Patients’ civil registration number, admitting hospital and department, dates of admission and discharge and diagnoses coded by physicians in charge are included in the records. All diagnoses are coded using the International Classification of Diseases (ICD) coding system [17] and with the replacement of the 8th edition, ICD8, with the 10th edition in 1995, ICD10 is used in this study. In our study population, only patients with a primary diagnosis of a first-time PE were included. The diagnosis could be registered initially at hospital admission or in the emergency department. Most diagnoses of cardiovascular disorders, including codes for PE, has a high validity (the ICD-code for a first-time PE has a positive predictive value of 88% in Danish registries) [18, 19]. Comorbidity was defined as any in-hospital diagnoses appearing within 5 years prior to the diagnosis of first-time PE in the DNPR. From the Central Person Register (CPR) we gathered information on all-cause 30-day mortality following the first-time PE diagnosis.

**Fig. 1:**
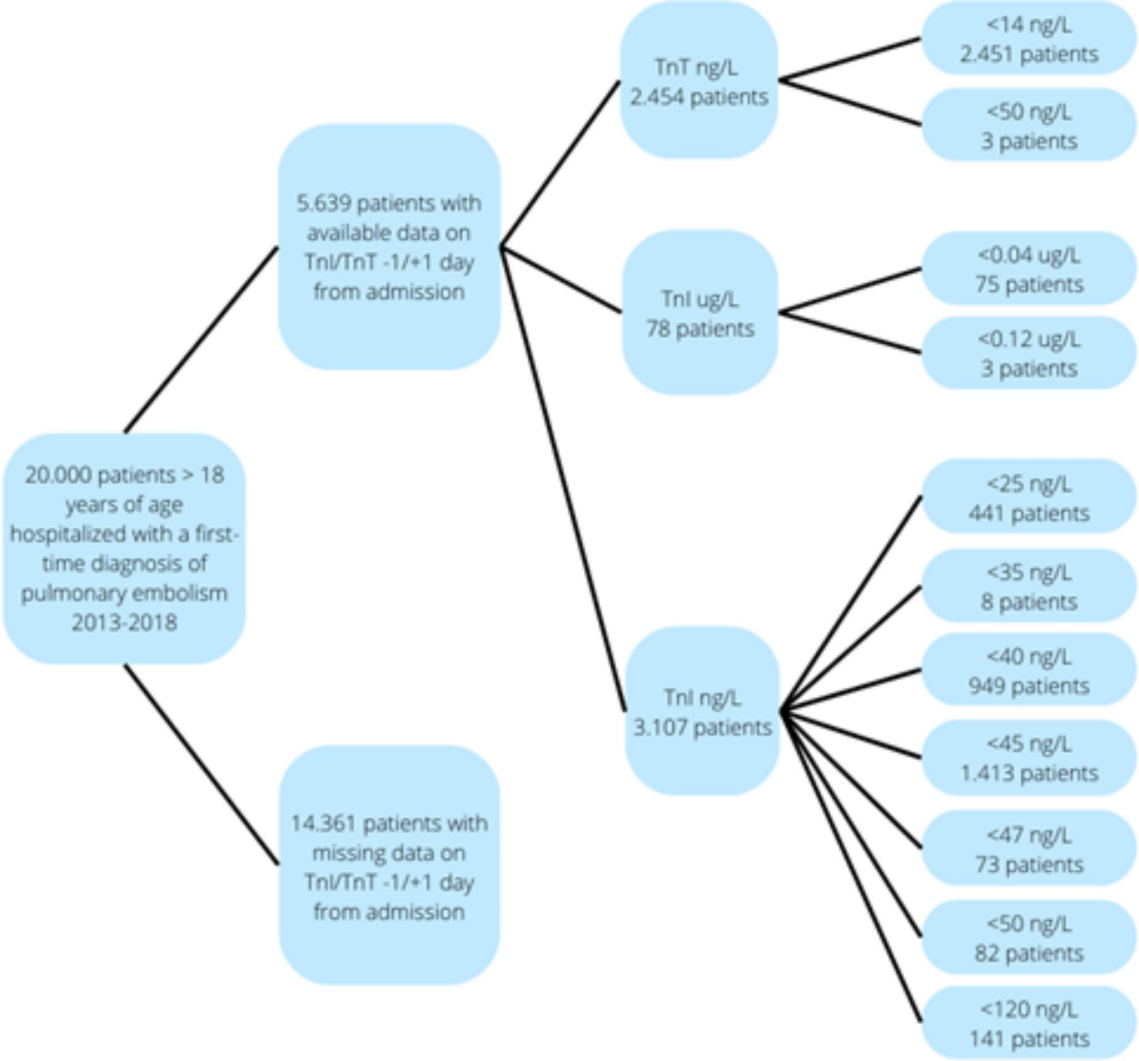
Flowchart showing data inclusion. The 11 identified patient groups based on different type of measurements (troponin I or T, unit and upper reference limit) are illustrated to the right

The clinical laboratory information system (LABKA) research database implemented in 1985 contains laboratory test results performed in private clinics and hospital departments covering most regions in Denmark since 2013 [20]. Information is recorded in a uniform way according to the international Nomenclature, Properties and Units (NPU) coding system. By linking PE patients identified from the DNPR to the LABKA research database, we retrieved information on levels of TnT and TnI measured within one day prior to one day after admission for first-time PE. We identified one group of patients with measurements of TnT in nanogram per litre (ng/L) and two different upper reference levels (< 14 ng/L and < 50 ng/L). A second patient group had measurements of TnT in microgram per litre (µg/L) completed with two different upper reference levels (< 0.05 µg/L and < 0.12 µg/L) and finally a third patient group had TnI in ng/L performed with 7 different upper reference levels (< 25 ng/L, < 35 ng/L, < 40 ng/L, < 45 ng/L, < 47 ng/L, < 50 ng/L and < 120 ng/L) (see Fig. 1). Unfortunately, our data did not allow any insights into the specific assay used.

### Statistical methods

Peak level of TnI and/or TnT per patient measured within one day prior to one day after admission for first-time PE were registered. Patients were dichotomized based on whether TnI/TnT concentration was elevated or not. Elevation was defined as a peak measurement with a concentration above individual upper reference level. Differences between patients with troponin above and under upper threshold with regards to median age, sex, comorbidities (ischemic heart disease (IHD), previous acute myocardial infarct (AMI), cancer, heart failure (HF), chronic obstructive pulmonary disease (COPD), renal disease, deep venous thrombosis (DVT)), median level of relevant biochemistry at time of PE diagnosis (haemoglobin (Hb), estimated glomerular filtration rate (eGFR) and C-reactive protein (CRP)) and 30-day mortality were examined using chi-squared for categorical variables and ANOVA tests for continuous variables.

We performed cumulative mortality curves and a log-rank test to compare differences in 30-day mortality from time of PE diagnosis between patients with elevated and non-elevated troponin. To investigate the individual effect of an elevated concentration of TnI/TnT on 30-day mortality we performed a cox proportional hazard model adjusting for age, sex, relevant pre-existing comorbidity (IHD, AMI, cancer, HF, COPD, renal disease and DVT) and biochemistry at time of PE diagnosis (Hb, eGFR and CRP). Concentrations of Hb, eGFR and CRP were divided into three levels (low, medium and high) based on tertiles.

Handling each assay separately, we further evaluated whether risk of 30-day mortality were proportional with the concentration of TnI/TnT, by dividing measurements into quintiles. We only included the eight different troponin analysis groups with > 50 patients each (see Fig. 1) and thus, the 5,625 troponin measurements were divided into five equal sized groups of approximately 1,100 patients by increasing concentration of troponin. Differences between each quintile regarding median age, sex, comorbidities and 30-day mortality were examined using Cochran Armitage Trend test for continuous variables and Mann Whitney U tests for categorical variables. Additionally, we assessed mortality using a cumulative mortality plot and a cox proportional hazard model adjusting for age, sex, and relevant pre-existing comorbidity to confirm differences in 30-day mortality between quintiles.

All dataset preparation was performed in SAS 9.4 and analyses and figures were performed in R 3.6.1. A two-sided p-value of < 0.05 was considered statistically significant.

### Ethics

This study was approved by the Danish Data Protection Agency (2007-58-0015, internal reference GEH-2014-015, I-suite: 02733). In Denmark, register studies do not require ethical approval. Data for this study was accessed via an encrypted server hosted by Statistics Denmark with anonymized data, and patients’ identities and sensitive information were thereby protected.

## Results

From 2013 to 2018 we identified 20,000 patients ≥ 18 years of age hospitalized with a first-time PE diagnosis in Denmark. No information on clinical parameters were available, and thus, patients were unselected regarding hemodynamic status. Among these patients, only 5,639 had a measurement of TnI (3,185 patients) or TnT (2,454 patients) completed within + 1/-1 day from admission (74% of measurements performed on the day of admission). Based on individual upper reference limit, 58% of patients (3,278 patients) had an elevated concentration of troponin, while 42% (2,361 patients) had a concentration within normal reference range (Table 1). There were no differences in the distribution of sex between the two patient groups (50% male in both). Patients with elevated troponin were older (median age 74 years) compared to those with normal troponin levels (median age 67 years) and had a greater burden of comorbidities (previous AMI (8 vs. 6%), cancer (18 vs. 15%), heart failure (8 vs. 6%), COPD (10 vs. 8%) and renal disease (5 vs. 3%)) except for a previous DVT, which were more frequently occurring in patients with non-elevated troponin level (15 vs. 19%). Patients with an elevated concentration of troponin had a significantly lower median level of eGFR (68 vs. 83 ml/min) and higher median level of CRP (35 vs. 25 mg/L) at time of PE diagnosis compared to those with normal concentrations of troponin, but both groups showed no difference in level of Hb (Table 1).

**Table 1:** Baseline results for patients with a first-time pulmonary embolism between 2013–2018 and elevated and non-elevated troponin levels respectively

	Elevated troponin level, n = 3,278	Non-elevated troponin level, n = 2,361	P-value
Male sex (%)	1,646 (50)	1,190 (50)	0.889
Median age (25th -75th percentile)	74 (65–82)	67 (54–75)	< 0.001
Ischemic heart disease (%)	491 (15)	337 (14)	0.461
Previous acute myocardial infarction (%)	275 (8)	145 (6)	0.002
Cancer (%)	593 (18)	360 (15)	0.005
Heart failure (%)	277 (8)	152 (6)	0.005
Chronic obstructive pulmonary disease (%)	343 (10)	190 (8)	0.002
Renal disease (%)	178 (5)	72 (3)	< 0.001
Deep venous thrombosis (%)	497 (15)	439 (19)	< 0.001
Median haemoglobin (25th -75th percentile)	8.4 (7.5–9.2)	8.5 (7.7–9.1)	0.135
Median estimated glomerular filtration rate (25th -75th percentile)	68 (51–85)	83 (68–90)	< 0.001
Median C-reactive protein (25th -75th percentile)	35 (14–70)	25 (7–62)	< 0.001

**Fig. 2:**
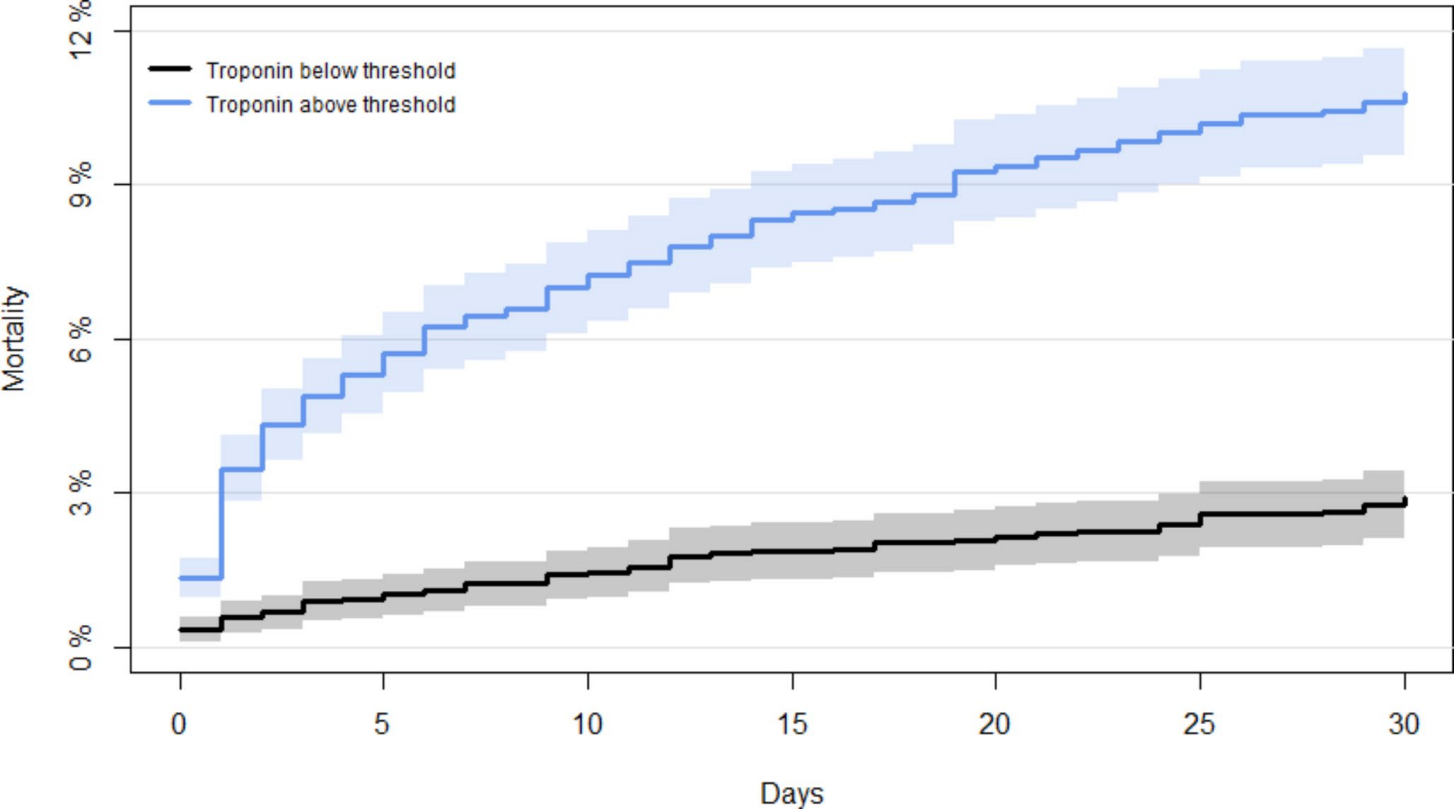
Cumulative mortality curve 0–30 days from first-time pulmonary embolism diagnosis. 30-day mortality differed significantly depending on troponin level below or above threshold, Log-rank test p < 0.001

30-day all-cause mortality following first-time PE differed between patients with elevated troponin and those with normal level, 11 vs. 3% respectively (Fig. 2) and was confirmed in an adjusted Cox regression model (HR_elevated_ troponin 2.74; 95% CI 1.94–3.86) even after adjustment for age, sex, comorbidities, Hb, eGFR and CRP (Table 2).

**Table 2:** Multivariable adjusted Cox proportional hazard model. CI: confidence interval

	Hazard ratio	95% CI		P-value
Troponin > threshold	2.74	[1.94 ; 3.86]		< 0.001
Male sex	1.02	[0.79 ; 1.32]		0.853
Age group				
18–44 years	0.49	[0.19 ; 1.27]		0.142
45–54 years	Ref.	Ref.		Ref.
55–64 years	0.86	[0.46 ; 1.61]		0.632
65–74 years	0.63	[0.32 ; 1.16]		0.138
>75 years	0.86	[0.47 ; 1.55]		0.609
Comorbidity				
Ischemic heart disease	1.02	[0.70 ; 1.48]		0.921
Previous acute myocardial infarction	1.13	[0.70 ; 1.85]		0.613
Cancer	1.88	[1.43 ; 2.46]		< 0.001
Heart failure	1.10	[0.72 ; 1.68]		0.655
Chronic obstructive pulmonary disease	1.54	[1.10 ; 2.14]		0.011
Renal disease	0.81	[0.49 ; 1.33]		0.397
Deep venous thrombosis	0.66	[0.44 ; 0.99]		0.045
Biochemistry				
Haemoglobin *High* *Medium* *Low*	Ref. 1.08 1.95	Ref. [0.74 ; 1.57] [1.38 ; 2.74]		Ref. 0.696 < 0.001
Estimated glomerular filtration rate				
*High* *Medium* *Low*	Ref. 0.95 1.88	Ref. [0.64 ; 1.41] [1.30 ; 2.71]		Ref. 0.811 < 0.001
C-reactive protein				
*High* *Medium* *Low*	Ref. 0.68 0.42	Ref. [0.51 ; 0.90] [0.29 ; 0.60]		Ref. 0.006 < 0.001

Comparing the five groups with increasing troponin concentrations, we found no difference in the distribution of male sex (1st quintile 49%, 2nd quintile 51%, 3rd quintile 54%, 4th quintile 51%, 5th quintile 46%). Median age increased with increasing concentration of troponin from 63 years in the 1st quintile group to 74 years in the 5th quintile group. The same was true for the proportion of patients with ischemic heart disease (1st quintile 11%, 2nd quintile 18%, 3rd quintile 18%, 4th quintile 15%, 5th quintile 12%) and heart failure (1st quintile 4%, 2nd quintile 8%, 3rd quintile 11%, 4th quintile 9%, 5th quintile 7%). Median concentration of eGFR decreased across quintiles (1st quintile 85 ml/min, 2nd quintile 81 ml/min, 3rd quintile 73 ml/min, 4th quintile 67 ml/min, 5th quintile 67 ml/min) whereas CRP increased (1st quintile 23 mg/L, 2nd quintile 24 mg/L, 3rd quintile 32 mg/L, 4th quintile 36 mg/L, 5th quintile 36 mg/L) (Table 3).

**Table 3:** Baseline results for patients with a first-time pulmonary embolism between 2013–2018 across quintiles of troponin measurement

	1st quintile, n = 1,107	2nd quintile, n = 1,107	3rd quintile, n = 1,110	4th quintile, n = 1,111	5th quintile, n = 1,115	P-value
Male sex (%)	545 (49)	563 (51)	595 (54)	567 (51)	517 (46)	0.629
Median age (25th -75th percentile)	63 (50–72)	70 (57–77)	74 (65–82)	74 (65–82)	74 (65–82)	< 0.001
Ischemic heart disease (%)	121 (11)	197 (18)	201 (18)	166 (15)	137 (12)	0.004
Previous acute myocardial infarction (%)	48 (4)	78 (7)	111 (10)	88 (8)	88 (9)	0.125
Cancer (%)	154 (14)	176 (16)	202 (18)	194 (17)	212 (19)	0.431
Heart failure (%)	42 (4)	83 (8)	119 (11)	105 (9)	77 (7)	0.039
Chronic obstructive pulmonary disease (%)	69 (6)	109 (10)	140 (13)	124 (11)	81 (7)	0.066
Renal disease (%)	26 (2)	33 (3)	52 (5)	64 (6)	73 (7)	0.065
Deep venous thrombosis (%)	220 (20)	201 (18)	177 (16)	165 (15)	152 (14)	0.492
Median haemoglobin (25th -75th percentile)	8.5 (7.8–9.1)	8.4 (7.5–9.1)	8.3 (7.5–9.1)	8.4 (7.6–9.1)	8.5 (7.5–9.2)	0.027
Median estimated glomerular filtration rate (25th -75th percentile)	85 (72–90)	81 (63–90)	73 (55–86)	67 (49–82)	67 (48–86)	< 0.001
Median C-reactive protein (25th -75th percentile)	23 (7–61)	24 (7–61)	32 (11–68)	36 (16–75)	36 (16–71)	< 0.001

**Fig. 3:**
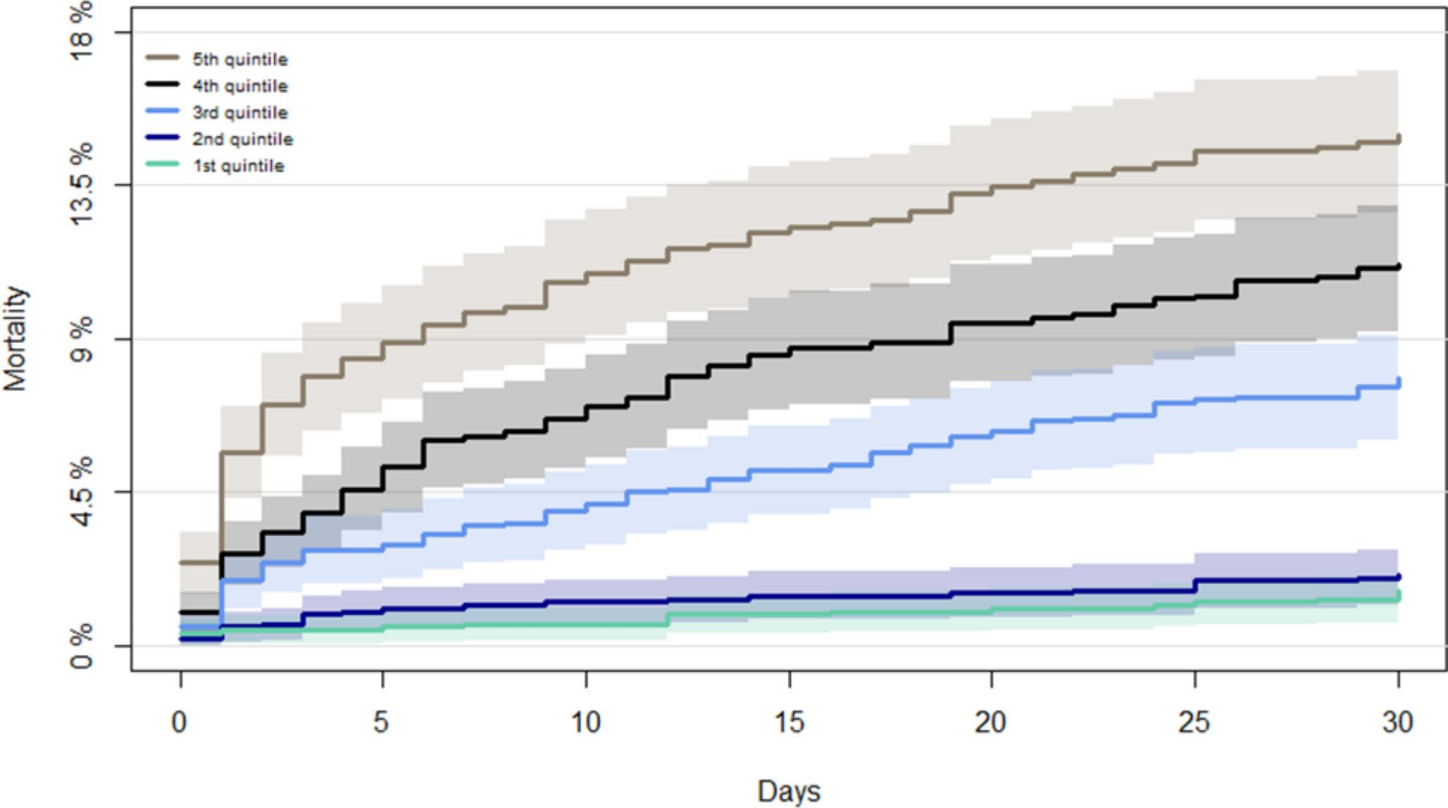
Cumulative mortality curve 0–30 days from first-time pulmonary embolism diagnosis. 30-day mortality differed significantly across troponin quintiles, Log-rank test p < 0.001

All-cause 30-day mortality following first-time PE varied across the quintile groups. No large difference in 30-day mortality was observed between 1st quintile group (1%) and 2nd quintile group (2%). However, 30-day mortality increased significantly across the remaining groups, from 8% in 3rd quintile group, 11% in 4th quintile group and 15% in 5th quintile group (Fig. 3).

A Cox regression model adjusted for age, sex, comorbidities and simultaneous Hb, eGFR and CRP revealed that 30-day mortality still increased across level of troponin, comparing 1st quintile with 2nd quintile (HR 0.84; 95% CI 0.36–1.95), 3rd quintile (HR 3.74; 95% CI 1.94–7.24), 4th quintile (HR 4.67; 95% CI 2.44–8.96) and 5th quintile (HR 6.55; 95% CI 3.46–12.38) respectively (Table 4).

**Table 4:** Multivariable adjusted Cox proportional hazard model. CI: confidence interval

	Hazard ratio	95% CI	P-value
Level of troponin			
1st quintile	Ref.	Ref.	Ref.
2nd quintile	0.84	[0.36 ; 1.95]	0.687
3rd quintile	3.74	[1.94 ; 7.24]	< 0.001
4th quintile	4.67	[2.44 ; 8.96]	< 0.001
5th quintile	6.55	[3.46 ; 12.38]	< 0.001
Male sex	1.04	[0.81 ; 1.35]	0.739
Age group			
18–44 years	0.50	[0.19 ; 1.30]	0.154
45–54 years	Ref.	Ref.	Ref.
55–64 years	0.78	[0.41 ; 1.47]	0.435
65–74 years	0.57	[0.31 ; 1.05]	0.071
>75 years	0.76	[0.42 ; 1.38]	0.360
Comorbidity			
Ischemic heart disease	1.08	[0.74 ; 1.56]	0.701
Previous acute myocardial infarction	1.10	[0.68 ; 1.79]	0.691
Cancer	1.89	[1.44 ; 2.48]	< 0.001
Heart failure	1.08	[0.71 ; 1.64]	0.726
Chronic obstructive pulmonary disease	1.61	[1.15 ; 2.25]	0.005
Renal disease	0.79	[0.48 ; 1.30]	0.348
Deep venous thrombosis	0.65	[0.43 ; 0.98]	0.038
Biochemistry			
Haemoglobin *High* *Medium* *Low*	Ref. 1.20 2.00	Ref. [0.82 ; 1.74] [1.42 ; 2.83]	Ref. 0.342 < 0.001
Estimated glomerular filtration rate			
*High* *Medium* *Low*	Ref. 0.96 1.78	Ref. [0.64 ; 1.42] [1.22 ; 2.59]	Ref. 0.821 0.002
C-reactive protein			
*High* *Medium* *Low*	Ref. 0.68 0.49	Ref. [0.52 ; 0.91] [0.34 ; 0.69]	Ref. 0.008 < 0.001

Treating the eight groups with > 50 patients and different upper threshold separately (Fig. 1), we found similar increasing associations between 30-day mortality and level of troponin (data not shown).

From the 20,000 identified patients with PE, 14,361 had non-available data on troponin measurements within − 1/+1 day from admission. Since the size of the missing data population is only partly due to the non-nationwide range of the LABKA database, we performed a sub analysis, revealing that age (median age 71 years in both) and sex (50% male in both) did not differ across patients with and without troponin measurements. More cancer patients were represented in the group with missing measurements of troponins (23% vs. 17%). The absolute risk of 30-day mortality was 8% in both patient groups (appendix table).

## Discussion

In this study of 5,639 unselected patients with acute PE, increasing troponin levels were associated with an increased 30-day mortality. Troponins were elevated in 58% of the patients studied and our results add, that increasing levels of troponin provides incremental prognostic information, which may be useful in selecting the appropriate therapy for PE patients.

This study agrees with previous findings, that a troponin measurement above threshold at time of PE-diagnosis increases the risk of 30-day mortality considerably [21, 22]. An elevated troponin measurement is associated with dysfunction of the right ventricle and more segmental defects in lung scans [22, 23] and thus represents a clinically important determinant of right ventricular overload. A meta-analysis from 2015 by Bajaj et al. including 16 studies on troponin levels in PE patients, found that overall, 11% of patients with elevated troponin levels and 3% of patients with normal levels died within 30 days [24]. Our results are in accordance with these outcomes. Another meta-analysis by Becattini et al. also concluded that the prognostic value of troponin was consistent among the 20 included studies regardless of the specific assay and relative cut-off point used [8]. The meta-analysis included works on both hemodynamic stable and unselected PE patients, as in our study.

An elevation of troponin concentration above a specific threshold, is thus included in the recommended risk stratification of PE patients to guide the choice of treatment strategy. With tachycardia and low systolic blood pressure resulting from a prognostic unfavourable acute RV failure, it is generally recognized that PE patients with unstable hemodynamics are to be treated with reperfusion treatment without the necessity of troponins to guide the therapeutic decision [1]. In opposition, hemodynamically stable PE patients need further risk assessment before decision on treatment approach. The spectrum of patients includes those with very low risk of death that could be managed in an outpatient clinic with novel oral anticoagulants. On the other side of the spectrum are patients with risk of poor outcomes that needs close monitoring and may benefit from more aggressive treatment, including thrombolytics. We had no data on hemodynamics in our patient population, and thus our results do not exclusively target the group of hemodynamic stable PE patients who needs further risk assessment. However, our study supports previous results, that TnI/TnT concentrations, independent of unit and upper reference limit, is an important independent marker of mortality risk in every PE patient [8]. Our baseline table comparing patients with and without registered troponin level at time of PE diagnosis revealed no significant difference in 30-day mortality.

The prognostic value of troponin measurements is considered most valuable when used in combination with echocardiography evaluating right ventricular function [10, 25]. Echocardiographic examination is not immediately available in all acute emergency rooms. Thus, it would be useful if the level of troponin solely could permit an extended early identification of patients at an increased risk of hemodynamic deterioration and death. In patients with unstable coronary artery disease it is known that the risk of cardiac events increases with increasing level of troponin in the first 24 hours [26]. However, in PE patients this possible incremental association between risk of death and increasing level of troponin concentrations is only rarely explored [22, 27]. In our study we found an increase of 30-day mortality with increasing concentration of troponins. Crude risk of death increased continuously from 1% in patients with troponins concentrations in the first quintile to 16% in those with concentrations in the fifth quintile and was confirmed in a multivariable Cox regression. No relevant difference in mortality was observed between first and second quintile group, however, mortality risk increased significantly and steadily across the third to the fifth quintile. This result is in accordance with a study by Konstantinides et al. from 2002, where measurements of TnI and TnT from 106 PE patients were divided into three groups representing low, moderate, and high concentrations. They found that the discrimination between moderate and pronounced elevation of troponin levels could be used to classify patients into an intermediate- and a high-risk group with regard to mortality and major clinical in-hospital events [22]. Another study by La Vecchio et al. including 48 patients with severe PE, concluded that a link between the degree of troponin increase and the severity of the clinical presentation exists. They observed no deaths among patients with normal concentrations, however, in-hospital mortality increased from 5% among those with slightly increased concentrations to 36% among those with still higher concentrations [27]. Lastly, a recent study by Ebner et al. confirmed the prognostic relevance of high-sensitive TnI in 459 normotensive PE patients and found, that patients who suffered an in-hospital adverse outcome had a significantly higher TnI concentration compared with those with a favourable clinical course [28]. Thus, our study including 5,608 PE patients, confirms these findings and suggests that not only a troponin concentration above threshold identifies PE at increased risk of early death, but also the exact level should be considered, when risk stratifying patients prior to a therapeutic decision. Additional clinical trials are needed to determine whether an extension of risk assessment depending on level of troponins are useful in guiding treatments of the spectrum of PE patients and improve prognosis of those in high risk of early mortality.

## Strengths and limitations

Due to the thorough registration practice in Denmark, our data cover almost the entire nation and as a result, our study population is one of the largest exploring the association between mortality and troponin concentration in patients with acute PE. In Denmark, the coding of diagnoses of cardiovascular disorders, including codes for PE, has a high validity [18, 19] making the register-based design of this study relevant in the discussion of the prognostic influence of troponins.

Due to the retrospective collection of data, our work does not contain information on other important prognostic risk factors, such as hemodynamic status and echocardiographic findings, that also contributes to the risk stratification of PE patients. Thus, our study results are not limited to the hemodynamic stable PE patients and we cannot evaluate whether there is an incremental prognostic value of troponin concentration in combination with echocardiographic findings.

In this study, the registration of expected peak level of troponin may be subject to some uncertainty. However, by choosing a time slot for peak measurement between − 1/+1 day from admission, we assume that we register most relevant peak concentration related to the PE-diagnosis.

Lastly, since different troponin analyses are used across Danish regions, standardization of troponin measurements was not possible in this study and limits the generalization of specific cut-off values. However, the demonstrated association between increasing risk of death and concentration of troponin is generalizable to every PE patient regardless type of troponin measurement used.

## Conclusion

The results of this large register-based study confirm the prognostic value of a troponin measurement above threshold at time of diagnosis in patients with acute PE. In addition, we demonstrate an increase in 30-day mortality risk with increasing concentration of troponin. This finding could contribute to further evolvement of risk stratification in PE patients essential in guiding therapeutic decisions but needs to be confirmed in future therapeutic trials.

## Electronic supplementary material

Below is the link to the electronic supplementary material.

Supplementary Material 1
