# Supplementary material for: Troponin dependent 30-day mortality in patients with acute pulmonary embolism
Source: J Thromb Thrombolysis. 2023 Jul 24;56(3):485–94. doi: 10.1007/s11239-023-02864-0 (PMC10439039; doi:10.1007/s11239-023-02864-0)
Supplement: Supplementary file 1 — Supplementary Material 1 [file 11239_2023_2864_MOESM1_ESM.docx]

|  | Troponin missing,  n = 14,361 | Troponin measured,  n = 5,639 | P-value |
| --- | --- | --- | --- |
|  |  |  |  |
| Male sex (%) | 7,084 (49) | 2,836 (50) | 0.211 |
|  |  |  |  |
| Median age (25^th^-75^th^ percentile) | 71 (60-79) | 71 (60-79) | 0.748 |
|  |  |  |  |
| Ischemic heart disease (%) | 1,989 (14) | 828 (15) | 0.127 |
| Acute myocardial infarct (%) | 931 (6) | 420 (8) | 0.014 |
| Cancer (%) | 3,231 (23) | 953 (17) | <0.001 |
| Heart failure(%) | 1,099 (8) | 429 (8) | 0.914 |
| Chronic obstructive pulmonary disease (%) | 1,525 (11) | 533 (9) | 0.015 |
| Renal disease (%) | 631 (4) | 250 (4) | 0.902 |
| Deep venous thrombosis (%) | 2,416 (17) | 936 (17) | 0.702 |
|  |  |  |  |
| Death within 30 days (%) | 1,193 (8) | 432 (8) | 0.060 |

**Appendix** **table** – Baseline table comparing patients with missing measurements of troponin and patients with registered measurements.
